# How might contact with nature promote human health? Promising mechanisms and a possible central pathway

**DOI:** 10.3389/fpsyg.2015.01093

**Published:** 2015-08-25

**Authors:** Ming Kuo

**Affiliations:** Landscape and Human Health Laboratory, Department of Natural Resources and Environmental Sciences, University of Illinois at Urbana-ChampaignUrbana, IL, USA

**Keywords:** natural environment, greenspace, immune, mechanism, mental health, literature review

## Abstract

How might contact with nature promote human health? Myriad studies have linked the two; at this time the task of identifying the mechanisms underlying this link is paramount. This article offers: (1) a compilation of plausible pathways between nature and health; (2) criteria for identifying a possible central pathway; and (3) one promising candidate for a central pathway. The 21 pathways identified here include environmental factors, physiological and psychological states, and behaviors or conditions, each of which has been empirically tied to nature and has implications for specific physical and mental health outcomes. While each is likely to contribute to nature’s impacts on health to some degree and under some circumstances, this paper explores the possibility of a central pathway by proposing criteria for identifying such a pathway and illustrating their use. A particular pathway is more likely to be central if it can account for the size of nature’s impacts on health, account for nature’s specific health outcomes, and subsume other pathways. By these criteria, *enhanced immune functioning* emerges as one promising candidate for a central pathway between nature and health. There may be others.

## Introduction

Contact with nature has been tied to health in a plenitude of studies. Time spent in and around tree-lined streets, gardens, parks, and forested and agricultural lands is consistently linked to objective, long-term health outcomes. The less green a person’s surroundings, the higher their risk of morbidity and mortality – even when controlling for socioeconomic status and other possible confounding variables. The range of specific health outcomes tied to nature is startling, including depression and anxiety disorder, diabetes mellitus, attention deficit/hyperactivity disorder (ADHD), various infectious diseases, cancer, healing from surgery, obesity, birth outcomes, cardiovascular disease, musculoskeletal complaints, migraines, respiratory disease, and others, reviewed below. Finally, neighborhood greenness has been consistently tied to life expectancy and all-cause mortality (see Table 3 in the Supplementary Materials).

These findings raise the possibility that such contact is a major health determinant, and that greening may constitute a powerful, inexpensive public health intervention. It is also possible, however, that the consistent correlations between greener surroundings and better health reflect self-selection – healthy people moving to or staying in greener surroundings. Examining the potential pathways by which nature might promote health seems paramount — both to assess the credibility of a cause-and-effect link and to suggest possible nature-based health interventions. Toward that end, this article offers: (1) a compilation of plausible pathways between nature and health; (2) criteria for identifying a possible central pathway; and (3) one promising candidate for a central pathway.

## How Nature Might Promote Health: Plausible Pathways

**FIGURE 1:**
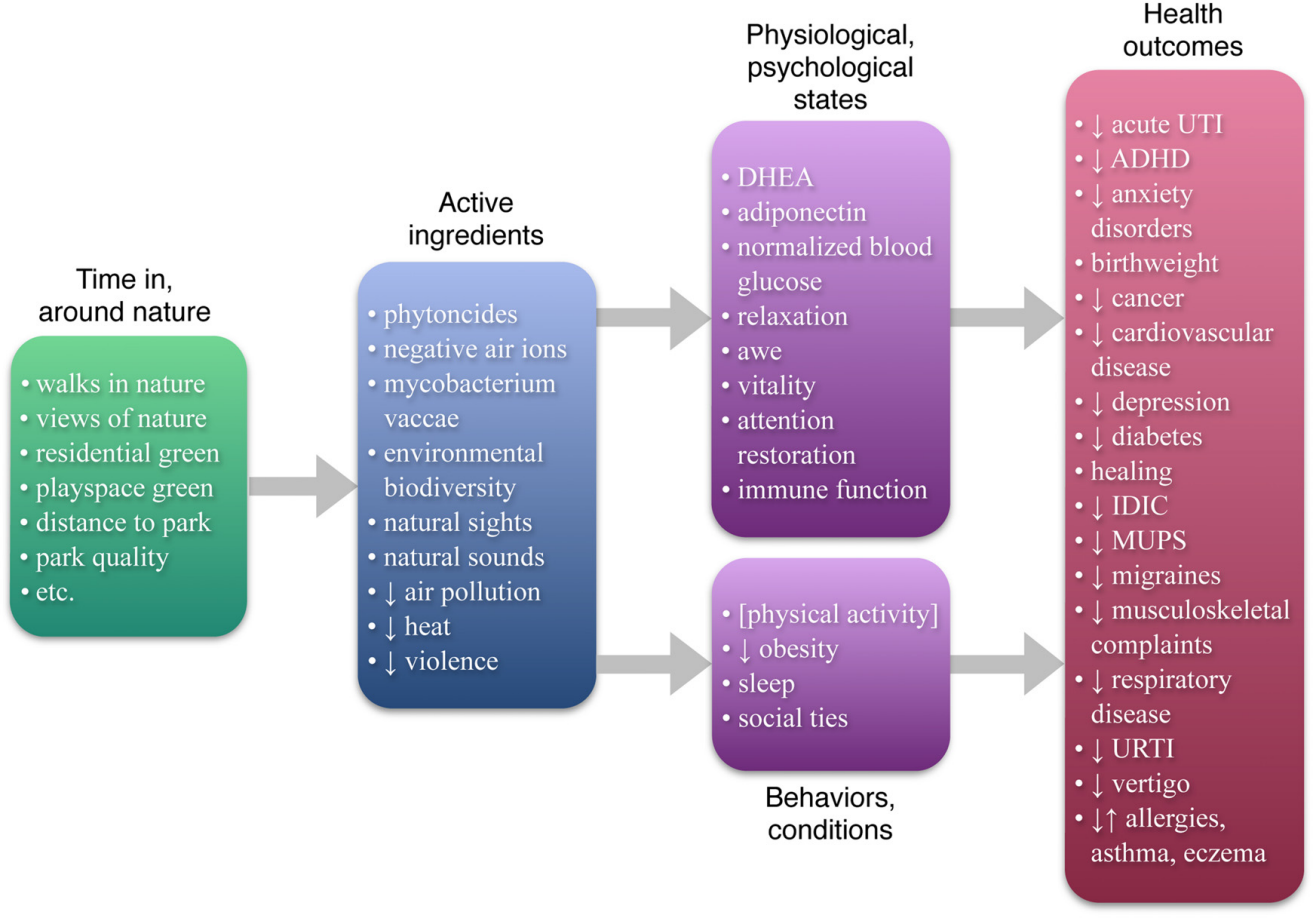
**The nature-health link: filling in the details**. This Figure summarizes the state of the scientific literature on nature and health, listing (1) the “active ingredients” in nature that have been identified as having impacts on health or health antecedents; (2) physiological/psychological states, behaviors, and conditions tied to both nature and health; and (3) specific health outcomes that have been tied to nature (controlling for socioeconomic variables). Note that physical activity (in brackets) is only sometimes tied to nature; and that allergies, asthma, and eczema are sometimes positively and sometimes negatively tied to nature. DHEA: didehydroepiandrosterone; acute UTI: acute urinary tract infection; ADHD: attention deficit hyperactivity disorder; IDIC: infectious disease of the intestinal canal; MUPS: medically unexplained physical symptoms; URTI: upper respiratory tract infection.

How might contact with nature promote health? To date, reviews and studies addressing multiple possible mechanisms (Groenewegen et al., 2006, 2012; Sugiyama et al., 2008; de Vries et al., 2013; Hartig et al., 2014) have focused on four – air quality, physical activity, stress, and social integration. But the burgeoning literature on nature benefits has revealed an abundance of possible mechanisms: as **Figure 1** shows, this review identifies 21 plausible causal pathways from nature to health. Each has been empirically tied to contact with nature while accounting for other factors, and is empirically or theoretically tied to specific health outcomes (for details on the scope of this review, see Table 1 in the Supplementary Materials). The 21 pathways identified here include environmental factors, physiological and psychological states, and behaviors or conditions, and are summarized below (for more details on each of these pathways, see Table 2 in the Supplementary Materials).

### Environmental Conditions

Some of the plausible pathways from contact with nature to improved health stem from specific environmental conditions. Natural environments contain chemical and biological agents with known health implications. Many plants give off phytoncides — antimicrobial volatile organic compounds — which reduce blood pressure, alter autonomic activity, and boost immune functioning, among other effects (Komori et al., 1995; Dayawansa et al., 2003; Li et al., 2006, 2009). The air in forested and mountainous areas, and near moving water, contains high concentrations of negative air ions (Li et al., 2010), which reduce depression (Terman et al., 1998; Goel et al., 2005), among other effects (Table 2 in the Supplementary Materials). These environments also contain mycobacterium vaccae, a microorganism that appears to boost immune functioning (see Lowry et al., 2007 for review). Similarly, environmental biodiversity has been proposed to play a key role in immune function via its effects on the microorganisms living on skin and in the gut, although the evidence for this is mixed (Table 2 in the Supplementary Materials).

The sights and sounds of nature also have important physiological impacts. Window views and images of nature reduce sympathetic nervous activity and increase parasympathetic activity (e.g., Gladwell et al., 2012; Brown et al., 2013), restore attention (e.g., Berto, 2005), and promote healing from surgery (Ulrich, 1984). Sounds of nature played over headphones increase parasympathetic activation (Alvarsson et al., 2010). These sympathetic and parasympathetic effects drive the immune system’s behavior (for review, see Kenney and Ganta, 2014), with long-term health consequences.

In built environments, trees and landscaping may promote health not only by contributing positive factors like phytoncides but also by reducing negative factors. Air pollution is associated with myocardial inflammation and respiratory conditions (Villarreal-Calderon et al., 2012). High temperatures can cause heat exhaustion, heat-related aggression and violence, and respiratory distress due to heat-related smog formation (Anderson, 2001; Akbari, 2002; Tawatsupa et al., 2012). And violence affects physical and mental health (e.g., Groves et al., 1993). Vegetation filters pollutants from the air (although see Table 2 in the Supplementary Materials for details), dampens the urban heat island (e.g., Souch and Souch, 1993), and appears to reduce violence (Table 2 in the Supplementary Materials for review).

### Physiological and Psychological States

Some of the plausible pathways between contact with nature and health involve short-term physiological and psychological effects, which, if experienced regularly, could plausibly account for long-term health effects.

Blood tests before and after walks in different environments reveal that levels of health-protective factors increase after forest but not urban walks. Didehydroepiandrosterone (DHEA) increases after a forest walk (Li et al., 2011); DHEA has cardio protective, anti-obesity, and anti-diabetic properties (Bjørnerem et al., 2004). Similarly, time in nature increases adiponectin (Li et al., 2011), which protects against atherosclerosis, among other things (Table 2 in the Supplementary Materials), and the immune system’s anti-cancer (so-called “Natural Killer,” or NK) cells and related factors (Table 2 in the Supplementary Materials). NK cells play important protective roles in cancer, viral infections, pregnancy, and other health outcomes (Orange and Ballas, 2006).

Further, walks in forested, but not urban areas, reduce the levels of health risk factors, specifically inflammatory cytokines (Mao et al., 2012), and elevated blood glucose (Ohtsuka et al., 1998). Inflammatory cytokines are released by the immune system in response to threat, and have been implicated in diabetes, cardiovascular disease, and depression (Table 2 in the Supplementary Materials). Chronically elevated blood glucose carries multiple health risks, including blindness, nerve damage, and kidney failure (Sheetz and King, 2002). The powerful effects of a walk in a forest on blood glucose are particularly striking (Table 2 in the Supplementary Materials for review).

Contact with nature has a host of other physiological effects related to relaxation or stress reduction (Table 2 in the Supplementary Materials). The experience of nature helps shift individuals toward a state of deep relaxation and parasympathetic activity, which improves sleep (El-Sheikh et al., 2013), boosts immune function in a number of ways (Kang et al., 2011), and counters the adverse effects of stress on energy metabolism, insulin secretion, and inflammatory pathways (Bhasin et al., 2013). Evidence suggests this pathway contributes substantially to the link between nature and health (Table 2 in the Supplementary Materials).

Three psychological effects of nature — experiences of awe (Shiota et al., 2007), enhanced vitality (Ryan et al., 2010), and attention restoration (Table 2 in the Supplementary Materials) — offer additional possible pathways between nature and health. Regular experiences of awe are tied to healthier, lower levels of inflammatory cytokines (Stellar et al., 2015); the ties between nature and awe, and awe and cytokines, respectively, may help explain the effects of forest walks on cytokines above. Similarly, feelings of vitality predict resistance to infection (Cohen et al., 2006) and lowered risk of mortality (Penninx et al., 2000). Attention restoration could theoretically reduce accidents caused by mental fatigue and, by bolstering impulse control, reduce risky health behaviors such as smoking, overeating, and drug or alcohol abuse (Wagner and Heatherton, 2010).

### Behaviors and Conditions

The remaining four possible pathways between contact with nature and health identified here involve behaviors and conditions: physical activity, obesity, sleep, and social ties. Physical activity is a major contributor to health (Centers for Disease Control and Prevention [CDC], 2015), and intuitively we associate green space with physical activity — but empirically this relationship is surprisingly inconsistent (Table 2 in the Supplementary Materials) and may hold only under certain conditions and for certain populations. Perhaps still more surprising, while greener residential areas do not consistently predict physical activity, they do consistently predict lower rates of obesity (for review, see Table 2 in the Supplementary Materials); this suggests the pathway between nature and obesity may depend less on nature’s effects on physical activity and more on its effects on adiponectin, stress, and impulse control. Both sleep and social ties are major contributors to health (Table 2 in the Supplementary Materials); contact with nature contributes to both better sleep (Morita et al., 2011; Astell-Burt et al., 2013) and stronger social ties (see Table 2 in the Supplementary Materials for review).

## Exploring the Possibility of a Central Pathway

Each of the mechanisms above is likely to contribute to nature’s impacts on health to some degree and under some circumstances. Most likely, some pathways will play a larger role than others. This paper explores the possibility that one or a few pathways may explain the lion’s share of the link between nature and health by proposing criteria for identifying central pathways and illustrating the application of these criteria.

First, a pathway is more likely to be central if it can account for the size of nature’s impacts on health. A study of over 345,000 people living in greener and less green residential surroundings revealed large differences in the prevalence of disease; even after controlling for socioeconomic status, prevalence for 11 major categories of disease was at least 20% higher among the individuals living in less green surroundings (Maas et al., 2009). For a single pathway to plausibly account for the bulk of the tie between nature and health, the mechanism involved would need to have substantial effects on health, and be substantially affected by contact with nature.

Second, a pathway is more likely to be central if it can account for specific health outcomes tied to nature. Although health is often treated as a unitary construct in the nature-health literature, poor health takes a multiplicity of separable, largely independent forms. A pathway that leads to one health outcome may not lead to others; for example, reduced air pollution may lessen respiratory symptoms, but is not likely to affect ADHD symptoms. A central pathway between nature and health should account for many, if not most, of the specific health outcomes tied to nature.

Third, a pathway is more likely to be central if it subsumes other pathways. To the extent that multiple nature-health pathways feed into a particular pathway between nature and health, that pathway is more central to the relationship between nature and health.

These three criteria can be applied to any given pathway to determine its centrality. In this paper, they are applied to one particular pathway: enhanced immune functioning.

### Criterion #1: Accounting for the Size of the Nature-Health Link

Determining whether a particular pathway can account for the size of the nature–health link requires examining the effect sizes for the nature–mechanism and mechanism–health relationships. For the immune system, the existing literature reveals both these effect sizes to be large.

Time spent in nature has substantial beneficial effects on the immune system, raising positive indicators, and lowering negative ones. Two 2-h forest walks on consecutive days increased the number and activity of anti-cancer NK cells by 50 and 56%, respectively, and activity remained significantly boosted even a month after returning to urban life — 23% higher than before the walks (Li, 2010). Moreover, extended time in a forest decreased inflammatory cytokines implicated in chronic disease by roughly one-half (Mao et al., 2012). Urban walks have no such effect.

The immune system, in turn, has powerful effects on health. The cytotoxic activity of NK cells is important in preventing cancer – in an 11-year study, the incidence of cancer among the individuals in the middle and top third of cytotoxic activity was roughly 40% less than that among individuals with low levels of NK activity (Imai et al., 2000). NK cells also play important health-promoting roles in fighting viral and other infections, in autoimmune disorders, and in pregnancy (see Orange and Ballas, 2006 for review). Moreover, inflammatory cytokines are thought to play an important role in a host of chronic diseases, including diabetes, cardiovascular disease, and depression (Cesari et al., 2003; Wellen and Hotamisligil, 2005; Dowlati et al., 2010).

It appears that enhanced immune function fulfills the first criterion for a central pathway: it can account for the size of nature’s apparent impacts on health.

### Criterion #2: Accounting for the Specific Health Outcomes Tied to Nature

Each specific health outcome tied to nature constitutes a testable hypothesis for any proposed central pathway; the more specific health outcomes a pathway can account for, the more central its role.

Contact with nature has been linked to a plethora of specific health outcomes; in general, the more contact with nature, the better the health outcome, even after controlling for socioeconomic status and other factors. For each of the following, available evidence points to a favorable impact: acute urinary tract infections, anxiety disorder, ADHD, birth outcomes, cancer, cardiovascular disease, depression, diabetes mellitus, healing from surgery, infectious disease of the intestinal canal, musculoskeletal complaints, medically unexplained physical symptoms (MUPS), migraines, upper respiratory tract infections, respiratory disease, and vertigo (for details, see Table 3 in the Supplemental Materials). For allergies, asthma, and eczema, the apparent impact of nature varies; depending on the specific measures used and the place, the relationships are positive, negative, or null (Table 3 in the Supplemental Materials).

**FIGURE 2:**
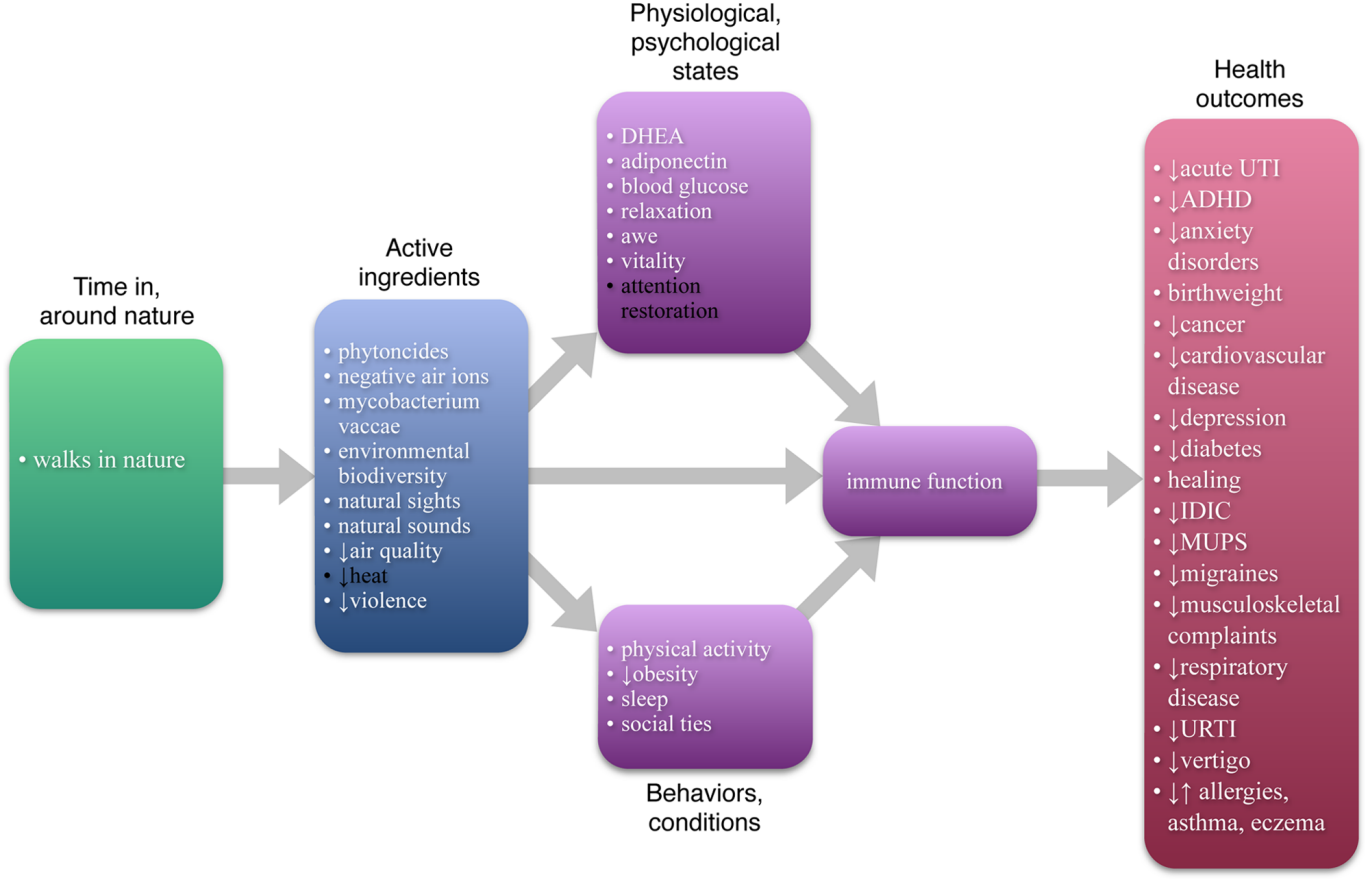
**Enhanced immune function as one possible central pathway**. All items in white text are currently known or have been proposed to be causally tied to immune function. As the Figure shows, enhanced immune function can account, at least partially, for each of the specific health outcomes currently tied to nature and may subsume, at least partially, all but two of the other pathways. Other pathways may also contribute the nature-health link, and other central pathways may exist.

To determine whether enhanced immune functioning could account for these specific health outcomes, the literature on immune functioning and each of the 18 specific outcomes was collected and reviewed. Available evidence indicates that enhanced immune functioning may be able to account, wholly or partially, for all 18 (see **Figure 2**).

One of the chief functions of the immune system is to ward off infectious disease, protecting the body from bacterial, parasitic, fungal, and viral infections (National Institutes of Health [NIH], 2012). Thus enhanced immune function could clearly explain why contact with nature is tied to lower rates of acute urinary tract infections, infectious disease of the intestinal canal, and upper respiratory tract infections. Further, for health outcomes with multiple possible origins, enhanced immune function can account for cases with infectious origins — for example, infectious forms of respiratory disease but perhaps not forms of respiratory disease with non-infectious origins. Similarly, enhanced immune function can account for MUPS and vertigo with infectious origins (Bovo et al., 2006; Deary et al., 2007, respectively).

Two other key roles of the immune system are to assist in wound healing, and to seek out and destroy tumor cells. Thus enhanced immune function can account for the effect of a hospital view of nature on recovery from surgery (Ulrich, 1984), as well as the relationship between residential greenness and lower rates of cancer (Li et al., 2010). Further, the immune system governs inflammation, which is involved in allergies (National Institutes of Health [NIH], 2015), anxiety disorder (Salim et al., 2012), asthma (Murdoch and Lloyd, 2010), cardiovascular disease (Mari et al., 2002; Ho et al., 2010; Schiffrin, 2013), depression (Calabrese et al., 2014), diabetes mellitus (Pedicino et al., 2013), eczema (National Institutes of Health [NIH], 2014), and musculoskeletal complaints (Ji et al., 2002; Wang et al., 2011). Finally, immune functioning is important in healthy birth weight (Moffett et al., 2014), and is suspected to play a role in ADHD (Segman et al., 2002; Budziszewska et al., 2010) and migraines (Bruno et al., 2007).

Available evidence indicates that enhanced immune function fulfills the second criterion for a central pathway.

### Criterion #3: Subsuming Other Pathways between Nature and Health

A nature–health pathway is more central if it subsumes other pathways; the more other pathways it subsumes, the more central its role. As **Figure 2** shows, the current literature suggests enhanced immune function can subsume as many as 18 out of the 20 other possible pathways between nature and health.

Enhanced immune function is known to wholly or partially subsume 11 other pathways. Each of the following is known to enhance immune function — adiponectin (Fantuzzi, 2013), reduced air pollution (e.g., Nadeau et al., 2010), awe (Stellar et al., 2015), normalized levels of blood glucose (as compared to elevated levels, Geerlings and Hoepelman, 1999), reduced obesity (de Heredia et al., 2012), physical activity (Shepherd et al., 1991), phytoncides (Li et al., 2009), better sleep (Besedovsky et al., 2012), social ties (Kiecolt-Glaser et al., 2002; Robles and Kiecolt-Glaser, 2003), relaxation and stress reduction (Abboud et al., 2012; Bhasin et al., 2013), and reduced immediate and long-term traumatic stress due to violence (e.g., Baum et al., 1993). Note that these pathways may be partially or wholly subsumed by the enhanced immune functioning pathway between nature and health; if a pathway contributes to health via both the immune system and other effects, it is partially subsumed by the immune function pathway.

For seven additional pathways, while there is no direct evidence tying them to human immune function, there is indirect evidence suggesting such a tie. DHEA (Hazeldine et al., 2010), mycobacterium vaccae (Lowry et al., 2007), and negative air ions (Yamada et al., 2006) are all known to improve immune function in mice. Vitality enhances resistance to upper respiratory tract infections (see vigor findings in Cohen et al., 2006), an effect mostly likely mediated via enhanced immune functioning. Both visual (e.g., Brown et al., 2013) and auditory (e.g., Alvarsson et al., 2010) nature stimuli are likely to boost immune function by way of their demonstrated effects on parasympathetic activity, and the subsequent effects of parasympathetic activity on immune function (Kenney and Ganta, 2014). And environmental biodiversity has been proposed to help train and regulate the immune system, although the findings here are correlational and mixed (e.g., Ruokolainen et al., 2014).

Enhanced immune function fulfills the third criterion for a central pathway.

## Conclusion

This review reveals a multiplicity of mechanisms by which contact with nature might promote health, as well as a promising candidate for a central pathway. There may be other mechanisms, such as other physiological effects, reduced accidents, and healthier behaviors. There may also be other central contributors to the nature-health link – of those reviewed here, deep relaxation, attention restoration and impulse control, sleep, and social ties seem particularly worthy of attention. No doubt some of the plausible pathways identified here will prove either not to contribute substantially to nature’s impact on health, or to contribute only under certain limited circumstances; here, the roles of improved air quality, environmental biodiversity and microbiota, and physical activity merit closer study.

These limitations notwithstanding, this review makes a number of contributions to our understanding of nature and health, to future investigation in this area, and to the creation of healthy human habitats. The multiplicity of nature–health pathways identified here lends credibility to the hypothesis that nature actually promotes health, as well as a potential explanation for the startling size and scope of nature’s apparent impact. With so many contributing pathways operating in concert, the cumulative effect could be quite large even if many of the individual pathways contribute only a small effect – that is, the effect of exposure to phytoncides *plus* exposure to mycobacteria vaccae *plus* increased adiponectin *plus* stronger social ties *plus* better sleep, etc., could indeed be quite large, and if some of the pathways, such as enhanced immune function, contribute a large effect, the combined effect would be larger still.

For future work in this area, the criteria here give researchers interested in central mechanisms a means of using existing literature to assess the centrality of a particular mechanism. In addition the detailed reviews provided in the Supplementary Materials may provide a useful starting point for researchers interested in specific pathways or specific health outcomes of nature.

Finally, the findings here can help guide the creation of healthy human habitats. The existing literature speaks to the value not only of “wild” nature but also “everyday” nature – the views and green spaces where we live. That physical activity is not consistently related to greener environments suggests that our conceptualization of health-promoting greenspaces should center at least as much on oases as on ball fields, and on greenspaces for walking and quiet contemplation as much as on recreation areas. The findings here suggest that such oases should incorporate plants — especially trees, soil, and water (preferably moving) — and should be designed to induce feelings of deep relaxation, awe, and vitality. Providing these green oases, especially in areas where health risks are high and landscaping is sparse, might be an inexpensive, powerful public health intervention and address persisting health inequalities.

## Conflict of Interest Statement

The author declares that the research was conducted in the absence of any commercial or financial relationships that could be construed as a potential conflict of interest.
