# Supplementary material for: How might contact with nature promote human health? Promising mechanisms and a possible central pathway
Source: Front Psychol. 2015 Aug 25;6:1093. doi: 10.3389/fpsyg.2015.01093 (PMC4548093; doi:10.3389/fpsyg.2015.01093)
Supplement: Supplementary file 1 [file Data_Sheet_1.DOCX]

## Table 1. Operational Definitions and Scope of Review

| **Construct** | **Criteria for inclusion** |
| --- | --- |
| Contact with Nature | *Contact with nature* here refers primarily to natural stimuli other than animals. These stimuli range from plants in an indoor setting, to built outdoor settings incorporating vegetation or water, to gardens, parks, and agricultural lands, to undeveloped natural and coastal areas and wilderness. Most studies in the nature benefits literature fall within two broad classes of exposure – background and event-based exposure.      The effects of background exposure to nature are studied in cross-sectional, epidemiological research. Typically, these studies compare health outcomes for individuals living with different levels of vegetation or green land cover within their residential environment. Greenness is measured in a variety of ways, from counts of street trees, to NDVI satellite measures of vegetation, to land cover types, to the presence and number of natural resource amenities. The unit of comparison varies in scale from the 1km radius around an individual’s home to the whole city; as the geographic units become larger, the correspondence between the amount of green cover and residents’ actual contact with nature is increasingly tenuous. Further, studies conducted at the largest scales tend to exclude the small-scale, near-home vegetation in yards and streetscapes from their measures of greenness. This is an important omission, as these forms of vegetation appear to be important contributors to health (e.g., de Vries et al 2013). Not surprisingly then, findings of nature-health links are more mixed at larger scales.      Experimental and quasi-experimental studies examine the immediate effects of event-based exposures; for example, they might measure blood pressure before and after a walk in a forest, and compare those findings with those from a walk in a pleasant but un-vegetated urban setting. |
| Health | *Health outcomes* were restricted for the purposes of this review to outcomes associated with medical diagnoses: symptomatology in patient populations as well as in the general population (e.g., depressive symptoms in patients with clinical depression as well as non-depressive patients); the prevalence of medically diagnosed diseases and disorders (e.g., the prevalence of cardiovascular disease); demands for care or treatment related to a particular disease (e.g., anti-anxiety medication prescriptions, general practitioner visits for musculoskeletal complaints); and mortality related to a particular health outcome (e.g. cancer-related mortality). Thus this review did not include general measures of perceived health or important forms of well-being such as life satisfaction and spiritual well being. |
| Mechanism | *Mechanism* (M) refers here to variables whose relationships to contact with nature (N) and health outcomes (HO) met the following criteria: (1) M was measured objectively (except for inherently subjective variables such as feelings of awe or vitality); (2) M had been theorized in previous literature to have causal ties to both nature and health, respectively, such that contact with nature would have effects on M which would then promote health; (3) the N–M link had been demonstrated empirically with controls for socioeconomic status; and (4) the M–HO link had been demonstrated empirically independent of its relationship to nature. Thus for example, the link between phytoncides and immune function was demonstrated by placing research participants in a hospital room and applying a small fan to a volatile gel of the fragrance of interest (e.g., Komori et al 1995). |
| Search for articles | “Snowball” sampling was used to identify empirical articles for possible inclusion in this review. The seed of the snowball was a Web of Science joint search for the terms “nature” and “health.” Empirical and review articles from peer-reviewed journals were retrieved if they addressed any of the pairwise relationships among contact with nature, health, and possible intervening mechanisms (as defined above). Each article so retrieved then served as the basis of a backwards and forwards search for additional articles, using the article’s Reference section and the “Cited Reference” function in Web of Science, respectively. This procedure was repeated for each new article identified until new searches yielded no new references. |

## Table 2. State of the literature: Mechanisms by which nature might promote health

How might spending time in and around nature promote health? Answering this question involves answering two broad subsidiary questions: What is it about nature that contributes to these effects –– what are the “active ingredients?” And what resulting changes in the individual then contribute to improved health? These health-promoting changes might be short-term physiological or psychological states, behaviors, or long-term conditions. The Table below lists plausible active ingredients and mediating variables identified in the existing scientific literature. For each, the Table presents a summary of available evidence regarding its possible tie to nature, and its significance for health promotion. Some of the evidence regarding these possible mechanisms has come from experimental or quasi-experimental work; other evidence comes from large-scale cross-sectional studies incorporating controls for socioeconomic status and other variables –– thus the causal tie between contact with nature and these possible mechanisms ranges from well-established to plausible. The existing research on one, oft-proposed, mechanism – physical activity – suggests that physical activity does not generally explain the tie between nature and health. Future research may rule out others of the possible mechanisms listed here, and may identify additional possible mechanisms.

| **Possible Mechanism** | **Ties to Nature** | **Health Significance** |
| --- | --- | --- |
| ***“Active ingredients”*** |  |  |
| Phytoncides | Forested, but not urban, environments have high concentrations of antimicrobial volatile organic compounds given off by plants, called phytoncides (Li et al 2010). | Phytoncides reduce blood pressure; increase parasympathetic nervous activity and decrease sympathetic nervous activity; boost immune functioning; and alleviate depressive states (Komori et al 1995; Dayawansa et al 2003; Li et al 2006, 2009). |
| Negative air ions | Forests, mountainous areas, and places with moving water have more negative air ions than urban settings – as much as four-fold higher (Mao et al 2012). | Negative air ions have systematic effects on serotonin, dopamine, and diastolic blood pressure (Ryushi et al 1998), and when administered regularly, alleviate depressive symptoms in chronic depression (Goel et al 2005) and Seasonal Affective Disorder (Terman et al 1998). Rigorous work with animal models suggests that negative air ions boost parasympathetic nervous activity and immune functioning (Suzuki et al 2008 and Yamada et al 2006, respectively). Numerous additional health benefits have been claimed for negative air ions, but the empirical basis for those claims is weak. |
| Mycobacterium vaccae | Mycobacterium vaccae are breathed in around soil, plants or water (Lowry et al 2007). | Mycobacterium vaccae have been found to boost the immune system via serotonin in mice (for review, see Lowry et al 2007). In metastatic malignant melanoma patients, adding mycobacterium vaccae injections to other interventions remarkably improves long-term survival (Cananzi et al 2013). |
| Environmental biodiversity | Environmental biodiversity has been proposed to contribute to human commensal microbiota –– the “good bacteria” living on or in the human body (von Hertzen et al 2011; Rook, 2013). There is some evidence consistent with this proposal: the more forest or agricultural land cover near a child’s home, the more proteobacteria on their skin and the more diverse their gammaproteobacteria (Hanski et al 2012; Ruokolainen et al 2014 has similar findings but does not control for socioeconomic status). Note that these findings did not hold for the diversity of vegetation in a child’s yard, nor for other bacterial classes on the skin (Hanski et al 2012). | Commensal microbiota are increasingly understood to play a role in the immune system’s ability to tolerate rather than attack non-threats. Consistent with this, the abundance of one particular commensal microorganism on the skin was correlated with levels of an anti-inflammatory cytokine playing a key role in immunologic tolerance (IL-10); this finding obtained in healthy individuals and not in atopic ones (Hanski et al 2012). |
| Nature Images | Most experiences of nature entail processing of visual natural stimuli. | As little as five minutes of exposure to images of trees, grass, and fields in a laboratory setting is enough to increase parasympathetic nervous activity and decrease heart rate (Gladwell et all 2012; Brown et al 2013). |
| Nature sounds | Many experiences of nature entail processing of natural sounds. | Exposure to nature sounds in a laboratory after a stressful mental arithmetic task speeds some aspects of physiological recovery relative to exposure to other sounds (Alvarsson et al 2010). Specifically, sounds of a fountain and of birds tweeting lowered skin conductance level – a measure of sympathetic nervous activity – more than did sounds of traffic or ambient building ventilation system sounds. However, heart rate variability – a measure of parasympathetic nervous activity – recovered no better during nature sounds than during other sounds (Alvarsson et al 2010). |
| Reduced air pollution | Vegetation filters both gaseous and particulate pollutants from the air (e.g., Fowler, 2002; Yoo et al, 2006). | By filtering pollutants from the air, vegetation may reduce the ill-effects of air pollution on myocardial inflammation, respiratory conditions, chronic obstructive pulmonary disease, and asthma (Villareal-Calderon et al 2012; Seguin 2008). However the helpful effects of vegetation on air pollution are small (Paoletti et al 2011, King et al 2014), and may be outweighed by the ill-effects of pollen (Lovasi et al 2013). |
| Reduced heat island effects | Trees, soil, and grass mitigate urban heat island effects (Souch & Souch 1993; Akbari 2002; Loughner et al 2012). | By mitigating the urban heat island effect, natural elements and spaces may reduce a variety of heat-related health risks, including heat exhaustion, heat stroke and sunstroke (e.g., Tawatsupa et al 2012); heat-related aggression and violence (Anderson 2001); and respiratory symptoms due to heat-related smog formation (Akbari 2002). |
| Reduced violence | Residential greenness has been tied to lower levels of aggression and violence (Kuo & Sullivan 2001a, b; Branas et al 2011; Wolfe & Mennis 2012; Troy et al 2012; cf Kondo et al 2015). Some evidence suggests the impact of nature on aggression is mediated by reduced mental fatigue, theoretically making individuals less irritable and less likely to speak or act impulsively (Kuo and Sullivan, 2001b). In addition, it is interesting to note that both the mycobacterium vaccae and negative air ions found in natural settings have been implicated in serotonin metabolism (Lowry et al 2007; Ryushi et al 1998), and that serotonin is believed to keep impulsive aggression in check (Seo et al 2008). | By reducing violence, residential greenness may reduce associated mental and physical harms to victims as well as mental harms to observers (e.g., Groves et al 1993). |
| ***Physiological and psychological states*** |  |  |
| DHEA | Walking in a forest, but not urban area, increases serum levels of didehydroepiandrosterone (DHEA) (Ohtsuka et al 1998). | DHEA has cardioprotective, anti-obesity, and anti-diabetic properties (Bjornerem et al 2004); thus regular forest walks could potentially protect against obesity, type 2 diabetes, hypertension, and coronary heart disease. |
| Adiponectin | Walking in a forest, but not urban area, increases serum levels of adiponectin (Li et al 2011). | Adiponectin has insulin sensitizing, anti-inflammatory, and anti-atherogenic properties (Simpson & Singh 2008); thus regular forest walks could potentially protect against obesity, type 2 diabetes, hypertension, and coronary heart disease. |
| Immune functioning | Walks in forested, but not urban, areas enhance immune functioning in two ways.  First, forest walks boost the number and activity of anti-cancer (so-called “natural killer” or NK) cells and the expression of anti-cancer proteins (Li et al 2007, 2008a, 2008b, 2010; Li 2010). Two 2-hour forest walks on consecutive days increased the number and activity of natural killer cells by 50% and 56%, respectively, and activity remained significantly boosted (23% higher) even a month after returning to urban life (Li 2010).  There is also evidence to suggest that forest, but not urban, walks reduce inflammatory cytokines (Mao et al 2012). | The immune system plays a central role in human health. Not only does it protect the body from bacterial, parasitic, fungal, and viral infections as well as toxins, drugs, and foreign particles, but it also seeks out and destroys tumor cells and plays an important role in many chronic diseases previously thought to be unrelated to immune functioning. NK cells play important protective roles in cancer, viral infections, pregnancy, and other health outcomes (Orange & Ballas, 2006), and inflammatory cytokines have been implicated in diabetes, cardiovascular disease, and depression (Cesari et al 2003; Wellen & Hotamisligil 2005; Dowlati et al 2010). Immune functioning is likely to be a major and even central contributor to nature’s effects on health. |
| Normalized blood glucose | Elevated blood glucose responds strongly to contact with nature. A relaxed, half hour walk in a forested area is sufficient to drop elevated blood glucose levels in diabetics 74 mg · 100 ml–1 (Ohtsuka et al, 1998) – roughly six times the benefit found for half an hour of indoor cycling in Schneider et al (1987). Further, for diabetics, a monthly routine of forest walks had substantial effects on mean blood glucose. The percentage of glycated haemoglobin A1c (HbA1c) in a person's blood reflects their mean blood glucose level in the previous 4–8 weeks, and 6.5% is the lowest level that qualifies as diabetic; monthly forest walks brought HbA1c levels down to 6.5% (Ohtsuka et al, 1998). | Persistently elevated blood glucose levels entail important health risks, including nerve damage, blindness, and kidney failure (Sheetz & King 2002); regular contact with nature might protect against these outcomes. |
| Relaxation and stress reduction | Exposure to nature has an array of physiological and psychological effects pertaining to relaxation and stress reduction. At one end of a continuum is a highly mobilized state known as “fight or flight;” at the other is a conscious but deeply relaxed state known as the “relaxation response;” and in between is baseline. By stimulating parasympathetic activity, a dose of nature can assist not only in restoring us to baseline from "fight or flight” but also move us past baseline to a state of deep relaxation (Gladwell et al 2012). Moreover, nature’s stress recovery effects are both retrospective and prospective – not only undoing lingering effects of past stressors (e.g., Ulrich, 1991), but preparing us to recover more quickly from future stressors (e.g., Brown et al 2013).      The psychological and physiological correlates of stress recovery encompass changes in mood as well as in cardiovascular, endocrine, and immune activity –– of these, perhaps the most well-studied in relation to nature are effects on cortisol, blood pressure, and heart rate (see Glaser & Kiecolt-Glaser, 2005, for review of the sympathetic-adrenomedullary and hypothalamic-pituitary-adrenocortical components of the stress response and its significance in health, and see Haluza et al 2014, for review of effects tied to nature). At the other end of the continuum, each of the following components of the relaxation response has been tied to nature:  parasympathetic dominance (e.g., Gladwell et al 2012), lower-than-baseline heart rate and blood pressure (Park et al, 2010), increases in alpha brain waves (Nakamura & Fuiji, 1990, 1992) and decreases in prefrontal cerebral activity (Park et al 2007; Horiuchi et al 2014). As the prefrontal cortex is the seat of directed attention and executive functioning, it seems likely that this reduction in cerebral activity is the physiological counterpart of the “respite from effortfully directed attention” described in the attention restoration literature as the basis for recovery from attention fatigue (Kuo 2001), and may be a precursor to attention restoration and vitality.       The relaxing and stress recovery effects of nature are large, fast, and sensitive to a wide variety of nature exposures: simply sitting in natural surroundings can increase parasympathetic activity by as much as 55% (Miyazaki et al 2014); substantial effects are seen with nature exposures of just 4-5 minutes (Brown et al 13; Gladwell et al 12; Ikei et al 14); and significant effects have been found not only for time spent in natural environments but also for visual only, smell only, tactile only, and sound only forest exposures (Miyazaki et al 2014). That the parasympathetic system is so responsive to even brief views of nature may help explain how residential greenness could have substantial impacts on health: regular micro-restorative experiences of natural views from home or the daily commute might help individuals achieve more relaxed states on an on-going basis; consistent with this idea, blood pressure and salivary cortisol are lower in populations with greener residential surrounds (Markevych et al 2014a; Ward Thompson et al 2012, respectively), as is sense of safety (Kuo et al 1998; Maas et al 2009). | Relaxation has important implications for health. Relaxation techniques, when used regularly, have documented, dose-response effects on immune functioning (Kang et al 2011). Deep states of relaxation counter the adverse effects of stress on energy metabolism, insulin secretion, and inflammatory pathways (Bhasin et al 2013), with potential implications for diabetes, cardiovascular disease, and other inflammatory disorders. Parasympathetic dominance appears to play an important role in sleep (El-Sheikh et al 2013). Interestingly, although stressful life events are consistently related to immune functioning, subjective reports of stress are not generally related to immune functioning (for review, see Segerstrom & Miller 2004).     Relaxation and stress recovery (or some aspect thereof) may play an important role in the link between nature and health. Four studies have examined the simultaneous relations among nature exposure, stressful circumstances or perceived stress, and health or well-being. Three found that greener residential surrounds reduced the impacts of stressful life events on measures of health or well-being (self-reported psychological distress in Wells & Evans 2003; perceived health in Van den Berg et al 2010; and mortality in Mitchell & Popham 2008). A fourth study examined residential greenness, perceived stress, and self-report measures of health, and found that the effects of greenness on perceived stress could account for a substantial portion of the greenness-health link (de Vries et al 2013). |
| Awe | Awe is sometimes experienced in response to nature, and may be experienced in response to nature as much as, or more than, it is experienced in response to music, art, or exceptional human achievements (Shiota et al 2007). | Regular experiences of awe predict lower, healthier levels of inflammatory cytokines; further, of all the positive emotions tested, awe showed the strongest link to healthier levels of inflammatory cytokines (Stellar et al 2015). |
| Vitality | Being outdoors in nature lends a sense of vitality -- energy available for purposive action (for review see Ryan et al 2010).     Although vitality has been investigated independently of attention restoration, it is likely that vitality and attention restoration are simply different facets of a single process. The descriptions of vitality (Ryan et al 2010) – energy available for purposive action –– sound very much like the descriptions of “rejuvenation” and “recovery from mental fatigue” associated with attention restoration (Kuo 2001). And multiple authors have proposed that attention restoration, state changes in executive functioning, and recovery from ego-depletion reflect the same underlying mechanisms (Kaplan & Berman 2010; Ryan et al 2010; Hofmann et al 2012); vitality may be the experiential component of these phenomena. | A sense of vitality predicts greater resistance to infection (see vigor findings in Cohen et al 2006) and lower risk of mortality (Penninx et al 2000). |
| Attention restoration | Contact with nature reduces mental fatigue and enhances impulse control (e.g., Kuo 2001; Faber Taylor et al 2002; Chow & Lau 2015). | Attention restoration could theoretically reduce fatigue-related accidents and risky health behaviors such as smoking, overeating, and drug or alcohol abuse (Wagner & Heatherton 2010). |
| ***Behaviors and conditions*** |  |  |
| Physical activity | Surprisingly, greener neighborhoods are not consistently linked with physical activity; the voluminous literature on green space and physical activity is quite mixed (for review, see Lachowycz & Jones 2011). Further, even in studies where physical activity is related to green, it does not appear to mediate the green-health relationship (Maas et al 2008; Lachowycz & Jones 2014). | Routine physical activity is important in weight control, mental health, and longevity, and reduces the risk of cardiovascular disease, Type 2 Diabetes, Metabolic Syndrome, and some cancers (CDC, 2011) |
| Obesity | Ten of ten studies have found evidence tying greener residential areas with lower rates of obesity (Liu et al 2007; Tilt et al 2007; Bell et al 2008; Potwarka et al 2008; Lovasi et al 2011; Wolch et al 2011; Lovasi et al 2012; Michimi & Wimberly 2012; Pereira et al 2013; Dadvand et al 2014). Residential greenness has been tied to lower rates of obesity across the lifespan, in rural and urban environments, for multiple – although not all – measures of greenness (park access, street trees, green cover, etc.) and for multiple measures of weight status (BMI, change in weight status, skin fold thickness).  There is some indication that the greenness-obesity relationship may be moderated by residential density (Liu et al 2007; Tilt et al 2007; Lovasi et al 2012). Perhaps related to this, greater variation of neighborhood greenness is linked to lower odds of obesity (Pereira et al 2013). | Obesity entails higher risks of many health problems, including cancer, coronary heart disease, type II diabetes, and stroke (NIH, 2012). |
| Sleep | Sleep troubles are eased after forest walks (Morita et al 2011), and short sleep is less common in greener residential surrounds (Astell-Burt et al 2013). | Inadequate sleep heightens risks for obesity (Chaput et al 2007; Cappuccio et al 2008), chronic disease (Heslop et al 2003; Cappuccio et al 2011), and mortality (Hublin et al 2007). |
| Social ties | A large body of studies ranging from experimental to large-scale epidemiological has tied greener settings to social variables, including but not limited to:  stronger pro-social aspirations and increased generosity (Weinstein et al 2009), greater shared use of common space and social interaction (Coley et al 1997; Faber Taylor et al 1998; Brunson et al 2001; Sullivan et al 2004), greater social safety (Maas et al 2009), greater mutual trust among neighbors and willingness to help one another (Cohen et al 2008), and greater social integration, social ties, and sense of community (Kuo et al 1998; Kweon et al 1998; Van den Berg et al 2010; Francis et al 2012; de Vries et al 2013; although c.f. mixed findings for different measures of green in Fan et al 2011). | Enhanced social ties appear to be a major contributor to nature’s effects on health. A voluminous literature attests to the importance of social ties for both mental and physical health (for review see Cohen, 2004; Holt-Lunstad et al 2010; Cacioppo & Cacioppo, 2014).     Moreover, findings from three studies point to a large role for social integration in the relationship between residential greenness and health. Each of these examined the relationships among residential greenness, social integration, and self-reported health in a large population, and employed statistical mediation testing (Baron & Kenny, 1986). Social integration substantially contributes to the relationship between greenness and perceived mental health (Sugiyama et al 2008; Maas, Van Dillen, et al 2009; de Vries et al 2013), perceived physical health (Sugiyama et al 2008); perceived general health (Maas, Van Dillen, et al 2009; de Vries et al 2013), and (fewer) health complaints (Maas, Van Dillen, et al 2009; de Vries et al 2013). No studies as of yet have examined whether these relationships hold for objective measures of health, such as mortality. |

## Table 3. Links between nature and specific health outcomes: State of the evidence

The literature on nature and “health” is now sufficiently developed that we can review the evidence for specific health outcomes. The Table below summarizes all findings pertaining to 18 specific health outcomes, whether positive, negative, or null. To provide as complete a picture for each specific health outcome as possible, a wide variety of evidence is brought to bear. All findings listed below incorporate controls for socioeconomic status.

| **Health outcome** | **Evidence regarding its relationship to contact with nature** |
| --- | --- |
| Acute urinary tract infections | Acute urinary tract infection is less prevalent among persons with a higher proportion of green land uses within 1 km of home (Maas et al 2009). |
| Anxiety disorder | Spending time in nature has positive short-term effects on mood (e.g., Song et al 2014); these effects appear to become “chronic” with regular nature exposure. Greener residential surrounds have been tied with lower levels of symptomatology for anxiety in the general population (Beyer et al 2014), lower prevalence of clinical anxiety disorders (Maas et al 2009), and fewer prescriptions for mood/anxiety treatments (Nutsford et al 2013). |
| Atopy (allergies, asthma, eczema) | Contact with nature, or more specifically, biodiversity, has been proposed to help the immune system learn to tolerate rather than attack non-threats (Rook, 2013). However, the findings on this question are extremely mixed, perhaps because vegetation has multiple effects –– capturing pollutants and training the immune system, on the positive side, as well as emitting pollen, on the negative. On the positive side, five studies find evidence that allergies, asthma and eczema – which all reflect hypersensitivity of the immune system –– are less prevalent among persons with greener residential surrounds (Lovasi et al 2008; Maas et al 2009 findings on asthma/chronic obstructive pulmonary disease; Hanski et al 2012; Fuertes et al 2014; Ruokolainen et al 2014). On the negative, four studies report exacerbated allergies or asthma in greener areas (DellaValle 2012; Lovasi et al 2013; Dadvand et al 2014; Fuertes et al 2014). And three studies find no effect (Maas et al 2009 findings on eczema; Dadvand et al 2014; Pilat 2012). |
| Attention-Deficit/Hyperactivity Disorder (ADHD) | Contact with nature appears to lead to both short- and long-term reductions in ADHD symptoms in children (Faber Taylor et al 2001; Kuo & Faber Taylor 2004; Faber Taylor & Kuo 2009, 2011; van den Berg & van den Berg 2011; Markevych et al 2014b). Such contact does not appear to affect the prevalence of ADHD (Baumgardner et al 2010). |
| Birth outcomes | Residential greenness during pregnancy is associated with healthier birth weights and lower risk of small-for-gestational-size, although this effect is sometimes stronger for or restricted to mothers of lower education (Donovan et al 2011; Dadvand et al 2012a, b, c; Hystad et al 2014). |
| Cancer | Nature has been tied to both short- and long-term outcomes related to cancer. Walks in forested but not urban areas boost the number and activity of anti-cancer (so-called "natural killer") cells and the expression of anti-cancer proteins (Li et al 2007, 2008a, b, 2010; Li 2010). Further, residential greenness predicts lower mortality rates for breast, uterine, and prostate cancer, although not all cancers (Li et al 2008). Note that Maas et al (2009) examined the relationship between residential greenness and cancer-related treatment in general practitioners' medical records and did not find a significant relationship; however it is not clear this null finding is meaningful, given that cancer care is provided by oncologists. Research on nature exposure and cancer should use oncologists' records to examine potential effects on prevalence, disease progression, and possible reductions in the side effects of chemotherapy on the immune system. |
| Cardiovascular disease | Contact with nature has been tied to both short- and long-term outcomes related to cardiovascular disease. Forest walks have a number of positive short-term effects on the cardiovascular system. Forest walks raise serum levels of adiponectin – which is antiatherogenic, and DHEA –which is cardioprotective (see table 2). In addition, in hypertensive patients, forest walks but not urban walks decrease serum levels of a number of factors associated with high blood pressure: endothelin-1, homocysteine, renin, angiotensin II type 1 receptor, and angiotensin II type 2 receptor (Mao et al 2012). Not surprisingly, then, forest but not urban walks lower blood pressure in young and middle-aged adults (Park et al 2010; Li et al 2011), as well as older adults with hypertension (Mao et al 2012).  These short-term effects, when experienced regularly, appear to promote cardiovascular health: individuals living in greener surrounds have lower blood pressure on average (Markevych et al 2014a), lower rates of cardiovascular disease (Maas et al 2009 for coronary heart disease, Pereira et al 2012, and Tamosiunas et al 2014), lower rates of cardiovascular mortality (Mitchell & Popham 2008; Coutts et al 2010; Richardson & Mitchell 2010; Villeneuve et al 2012; Donovan et al 2013), and higher survival rates after ischemic stroke (Wilker et al 2014). A handful of studies, generally comparing larger geographical units, find no relationship between greener areas and cardiovascular outcomes in one or more analyses (Coutts et al 2010, Richardson & Mitchell 2010; Richardson et al 2010, 2011). These exceptions notwithstanding, the preponderance of the evidence supports a link between contact with nature and cardiovascular health. |
| Depression | Greener residential surrounds have been tied with lower levels of depressive symptomatology in the general population (e.g., clinically non-depressed) (Beyer et al 2014), lower prevalence of clinical depression (Maas et al 2009), and fewer prescriptions for mood/anxiety treatments (Nutsford et al 2013). Note that Miles et al (2012) found that a larger acreage of near-home green spaces was linked to fewer depressive symptoms but this relationship became nonsignificant when income and other factors were controlled. |
| Diabetes Mellitus | Brief forest exposures have a number of short-term effects which suggest that regular nature exposure could improve diabetes outcomes –– stimulating the release of anti-diabetic hormones adiponectin and DHEA (Table 2), modulating insulin by way of its effects on parasympathetic activity (Bhasin et al 2013), and normalizing elevated blood glucose (Table 2). Indeed, in diabetics, monthly forest walks were sufficient to reduce glycated A1c to, on average, 6.5 – the threshold value for a diabetes diagnosis. Not surprisingly then, diabetes mellitus (Type 1 or 2) is less prevalent among individuals living in greener surrounds (Maas et al 2009; Astell-Burt et al 2014) and among park-users than non-park users (Tamosiunas et al 2014), although these findings do not obtain for some measures of greenness (Tamosiunas et al 2014) or at the city scale (Richardson et al 2011). |
| Healing from surgery | Patients in hospital rooms with green views recover faster than their counterparts facing a brick wall (Ulrich, 1984). |
| Infectious disease of the intestinal canal | Infectious disease of the intestinal canal was less prevalent among persons with a higher proportion of green land uses within 1 km of home (Maas et al 2009). |
| Medically unexplained physical symptoms | Medically unexplained physical symptoms were less prevalent among persons with a higher proportion of green land uses within 1 km of home (Maas et al 2009). |
| Migraines | Migraines are less prevalent in persons with a higher proportion of green land uses within 1 km of home (Maas et al 2009). |
| Musculoskeletal complaints | Musculoskeletal complaints were less prevalent among persons with a higher proportion of green land uses within a 1 km radius of home (Maas et al 2009). This finding obtained for each of the specific categories of musculoskeletal complaints: neck and back complaints; severe back complaints; severe neck and shoulder complaints; and severe elbow, wrist, and hand complaints. |
| Respiratory disease | Respiratory disease and related mortality are less prevalent in greener residential surrounds in four of four studies, although not in all analyses (Maas et al 2009; Richardson & Mitchell, 2010; Villeneuve et al 2012; Donovan et al 2013). |
| Upper respiratory tract infections | Upper respiratory tract infections were less prevalent among persons with a higher proportion of green land uses within 1 km of home (Maas et al 2009). |
| Vertigo | Vertigo was less prevalent among persons with a higher proportion of green within 1 km of home (Maas et al 2009). |
| All-cause mortality | Multiple epidemiological studies have examined life expectancy or mortality, with a preponderance of positive findings. A study of the over 3,000 counties in the U.S. found that those counties with higher percentages of land in forests, farmland, rangeland, and water bodies also had higher life expectancies at birth, controlling for a host of potential confounding factors (Poudyal et al 2009). Similarly, mortality rates are lower for greener areas in Tokyo (Takano, Nakamura et al 2002), greener Least Statistical Output Areas in the United Kingdom (Mitchell & Popham 2008), and greener metropolitan areas in the Netherlands (Jonker et al 2014). Note, however, some mixed findings. Lachowycz & Jones (2014) found a relationship between greenspace access and reduced mortality only in the most deprived areas. Takano, Fu et al (2002) found that the ward-units in Shanghai with a greater proportion of green land uses also had lower age-adjusted mortality rates, although this finding did not persist when extensive controls were included in the model. And a study comparing whole cities in the US found that, among the 49 largest US cities, those cities with the highest proportion of green space coverage had higher, not lower, rates of all-cause mortality (Richardson et al 2011). |

REFERENCES CITED IN TABLES

Note: references previously cited in the manuscript itself are listed in the corresponding Reference section.

Astell-Burt T, Feng XQ, & Kolt GS. (2014). Is neighborhood green space associated with a lower risk of type 2 diabetes? Evidence from 267,072 Australians. Diabetes Care, 37, 197-201.

Baron, RM, & Kenny, DA. (1986). The moderator-mediator variable distinction in social psychological research: conceptual, strategic and statistical considerations. Journal of Personality and Social Psychology, 51, 1173e1182.

Baumgardner, D. J., Schreiber, A. L., Havlena, J. A., Bridgewater, F. D., Steber, D. L., & Lemke, M. A. (2010). Geographic analysis of diagnosis of attention-deficit/ hyperactivity disorder in children: Eastern Wisconsin, USA. The International Journal of Psychiatry in Medicine, 40, 363-382.

Bell, J., Wilson, J., Liu, G. (2008). Neighborhood Greeness and 2-Year Changes in Body Mass Index of Children and Youth. Am J Prev Med; 35(6): 547-553.

Beyer, K.M.M., Kaltenbach, A., Szabo, A., Bogar, S., Javier Nieto, F., Malecki, K.M. (2014). Exposure to neighborhood green space and mental health: Evidence from the survey of the health of Wisconsin. International Journal of Environmental Research and Public Health, 11(3),3453-3472. doi:10.3390/ijerph110303453

Branas, C. C., Cheney, R. A., MacDonald, J. M., Tam, V. W., Jackson, T. D., & Ten Have, T. R. (2011). A difference-in-differences analysis of health, safety, and greening vacant urban space. American Journal of Epidemiology, 174, 1-11.

Brunson, L.B., Kuo, F.E., & Sullivan, W.C. (2001). Resident appropriation of defensible space in public housing: Implications for safety and community. Environment & Behavior 33(5), 626-652.

Cacioppo JT, & Cacioppo S. (2014). Social Relationships and Health: The Toxic Effects of Perceived Social Isolation. Social and Personality Psychology Compass, 8(2):58-72.

Cananzi FCM, Mudan S, Dunne M, Belonwu N, & Dalgleish AG. (2013). Long-term survival and outcome of patients originally given Mycobacterium vaccae for metastatic malignant melanoma. Human Vaccines & Immunotherapeutics 9:11, 2427-2433.

Cappuccio FP, Cooper D, D Elia L, et al. (2011). Sleep duration predicts cardiovascular outcomes: a systematic review and meta-analysis of prospective studies. Eur Heart J;32:1484–92.

Cappuccio FP, Taggart FM, Kandala NB, et al. (2008). Meta-analysis of short sleep duration and obesity in children and adults. Sleep;31:619–26.

Chaput JP, Després J-P, Bouchard C, et al. (2007). Association of sleep duration with type 2 diabetes and impaired glucose tolerance. Diabetologia;50:2298–304.

Chow JT & Lau S. (2015). Nature gives us strength: Exposure to nature counteracts ego-depletion. J Soc Psych 155(1):70-85. DOI: 10.1080/00224545.2014.972310

Cohen S. (2004). Social relationships and health. Am Psychol 2004, 59:676-84.

Coley, R.L., Kuo, F.E., & Sullivan, W.C. (1997). Where does community grow? The social context created by nature in Urban Public Housing. Environment and Behaviour 29 (4), 468–494.

Coutts, C., Horner, M., & Chapin, T. (2010). Using geographical information system to model the effects of green space accessibility on mortality in Florida. Geocarto International, 25, 471-484.

Dadvand P, de Nazelle A, Figueras F, Basagaña X, Sue J, Amoly E, et al. 2012a. Green space, health inequality and pregnancy. Environ Int 40:110–115.

Dadvand P, de Nazelle A, Triguero-Mas M, Schembari A, Cirach M, Amoly E, et al. 2012b. Surrounding greenness and exposure to air pollution during pregnancy: an analysis of personal monitoring data. Environ Health Perspect 120:1286–1290; doi: 10.1289/ehp.1104609.

Dadvand P, Sunyer J, Basagaña X, Ballester F, Lertxundi A, Fernández-Somoano A, et al. 2012c. Surrounding greenness and pregnancy outcomes in four Spanish birth cohorts. Environ Health Perspect 120:1481–1487; doi: 10.1289/ehp.1205244.

Dadvand, P., Villanueva, C. M., Font-Ribera, L., Martinez, D., Basagaña, X., Belmonte, J., ... & Nieuwenhuijsen, M. J. (2014). Risks and benefits of green spaces for children: A cross-sectional study of associations with sedentary behavior, obesity, asthma, and allergy. Environmental Health Perspectives,122,1329-1335.

DellaValle CT, Triche EW, Leaderer BP, Bell ML. 2012. Effects of ambient pollen concentrations on frequency and severity of asthma symptoms among asthmatic children. Epidemiology 23(1):55–63.

Donovan GH, Butry DT, Michael YL, Prestemon JP, Liebhold AM, Gatziolis D, & Mao MY. (2013). The relationship between trees and human health: Evidence from the spread of the Emerald Ash Borer. Am J Prev Med, 44, 139-145. doi: 10/1016/j.amepre.2012.09.066

Donovan, GH, Michael YL, Butry DT, Sullivan AD, & Chase JM. (2011). Urban trees and the risk of poor birth outcomes. Health and Place. 17:390-393.

Faber Taylor A, Kuo FE, & Sullivan WC. (2002). Views of nature and self-discipline: Evidence from inner city children. Journal of Environmental Psychology, 22, 49-63.

Faber Taylor A, Kuo FE, Sullivan WC. (2001). Coping with ADD: The surprising connection to Green Play Settings. Environment & Behavior, 33(1):54-77.

Faber Taylor, A., Kuo, F.E. (2009). Children with attention deficits concentrate better after walk in the park. Journal of Attention Disorders, 12(5), 402-409.

Faber Taylor, A., Kuo, F.E.M. (2011). Could exposure to everyday green spaces help treat adhd? Evidence from children's play settings. Applied Psychology: Health and Well-Being, 3(3), 281-303.

Faber Taylor, A., Wiley, A., Kuo, F.E., & Sullivan, W.C. (1998). Growing up in the inner city: Green spaces as places to grow. Environment & Behavior, (30)1, 3-27.

Fan, Y., Das, K., & Chen, Q. (2011). Neighborhood green, social support, physical activity, and stress: assessing the cumulative impact. Health & Place, 17, 1202e1211.

Fowler D. 2002. Pollutant deposition and uptake by vegetation. In Air Pollution and Plant Life, ed. JNB Bell, M Treshow, pp. 43–67. New York: Wiley. 2nd ed.

Francis J, Giles-Corti B, Wood L, Knuiman M. 2012. Creating sense of community: the role of public space. J. Environ. Psychol. 32:401–9

Fuertes, E., Markevych, I., von Berg, A., Bauer, C. P., Berdel, D., Koletzko, S., ... & Heinrich, J. (2014). Greenness and allergies: Evidence of differential associations in two areas in Germany. Journal of Epidemiology and Community Health, 68, 787-790.

Gaudillière, B., Fragiadakis, G. K., Bruggner, R. V., Nicolau, M., Finck, R., Tingle, M., ... & Nolan, G. P. (2014). Clinical recovery from surgery correlates with single-cell immune signatures. *Science Translational Medicine*, *6*, 255ra131-255ra131. DOI: 10.1126/scitranslmed.3009701

Glaser R, & Kiecolt-Glaser JK. (2005). Stress-induced immune dysfunction: implications for health. Nature Reviews: Immunology, 5, 243-251. DOI: 10.1038/nri1571

Haluza D, Schonbauer R, & Cervinka R. (2014).  Green Perspectives for Public Health: A Narrative Review on the Physiological Effects of Experiencing Outdoor Nature.  *Int. J. Environ. Res. Public Health*, *11*, 5445-5461; doi:10.3390/ijerph110505445

Hanski, I., von Hertzen, L., Fyhrquist, N., Koskinen, K., Torppa, K., Laatikainen, T., ... & Haahtela, T. (2012). Environmental biodiversity, human microbiota, and allergy are interrelated. Proceedings of the National Academy of Sciences, 109, 8334-8339.

Hanyu, K., Tamura, K., & Mori, H. (2014). Changes in heart rate variability and effects on POMS by whether or not soil observation was performed. Open Journal of Soil Science, 4, 36-41. doi 10.4236/ojss.2014.41005

Heslop P, Smith GD, Metcalfe C, et al. Sleep duration and mortality: the effect of short or long sleep duration on cardiovascular and all-cause mortality in working men and women. Sleep Med 2003;3:305–14.

Hofmann, W., Schmeichel, B. J., & Baddeley, A. D. (2012). Executive functions and self-regulation. Trends in Cognitive Sciences, 16, 174-180.

Holt-Lunstad J, Smith TB, Layton JB. 2010. Social relationships and mortality risk: a meta-analytic review. PLoS Med. 7:e1000316

Horiuchi M, Endo J, Takayama N, Murase K, Nishiyama N, Saito H, Fujiwara A.  (2014).  Int J Environ Res Public Health 11, 10883-10901. doi: 10.3390/ijerph111010883

Hublin C, Partinen M, Koskenvuo M, et al. (2007). Sleep and mortality: a population-based 22-year follow-up study. Sleep;30:1245–53.

Hystad P, Davies HW, Frank L, Van Loon J, Gehring U, Tamburic L, & Brauer M. (2014). Residential Greenness and Birth Outcomes: Evaluating the Influence of Spatially Correlated Built-Environment Factors. Environmental Health Perspectives, 122(10):1095-1102.  DOI: 10.1289/ehp.1308049

Ikei H, Song C, Kagawa T, & Miyazaki Y. (2014). Physiological and psychological effects of viewing forest landscapes in a seated position in one-day forest therapy experimental model. Nihon Eiseigaku Zasshi, 69, 104-10.

Jonker, M. F., van Lenthe, F. J., Donkers, B., Mackenbach, J. P., & Burdorf, A. (2014). The effect of urban green on small-area (healthy) life expectancy. Journal of Epidemiology and Community Health,68:999-1002. doi:10.1136/jech-2014-203847

Kaplan S, Berman MG. (2010). Directed attention as a common resource for executive functioning and self-regulation. Perspectives on Psychological Science 5(1):43-57. doi:10.1177/1745691609356784

King KL, Johnson S, Kheirbek I, Lu JWT, & Matte T. (2014). Differences in magnitude and spatial distribution of urban forest pollution deposition rates, air pollution emissions, and ambient neighborhood air quality in New York City. Landscape and Urban Planning, 128, 14–22. doi:10.1016/j.landurbplan.2014.04.009

Koivisto VA, DeFronzo RA (1984) Exercise in the treatment of type II diabetes. Acta Endocrinol [Suppl] 206:107–111.

Kondo MC, Low SC, Henning J, & Branas CC. (2015). The Impact of Green Stormwater Infrastructure Installation on Surrounding Health and Safety. American Journal of Public Health, 105(3), 114–121. doi:10.2105/AJPH.2014.302314

Kuo FE, Faber Taylor A. (2004). A potential natural treatment for Attention-Deficit/Hyperactivity Disorder: Evidence from a National Study. Am J Public Health, 94(9):1580-1586.

Kuo FE, Sullivan WC, Coley RL, & Brunson L. (1998). Fertile ground for community: Inner-city neighborhood common spaces. American Journal of Community Psychology, 26(6), 823-851

Kuo, FE (2001). Coping with Poverty: Impacts of environment and attention in the inner city. Environment & Behavior, 33(1), 5-34.

Kuo, FE, & Sullivan, WC. (2001a). Environment and crime in the inner city: Does vegetation reduce crime? Environment & Behavior, 33(3), 343-367.

Kuo, FE, & Sullivan, WC. (2001b). Aggression and Violence in the Inner City: Effects of Environment via Mental Fatigue. Environment & Behavior, Special Issue, 33(4), 543-571.

Kweon, B.S., Sullivan, W.C., Wiley, A. (1998). Green common spaces and the social integration of inner-city older adults. Environment and Behavior 30, 823–858.

Lachowycz K, Jones AP. 2011. Greenspace and obesity: a systematic review of the evidence. Obes. Rev. 12:e183–89

Lachowycz, K., & Jones, A. P. (2014). Does walking explain associations between access to greenspace and lower mortality?. Social Science & Medicine, 107, 9-17.

Li, Q., Morimoto, K., Kobayashi, M., Inagaki, H., Katsumata, M., Hirata, Y., ... & Krensky, A. M. (2008a). Visiting a forest, but not a city, increases human natural killer activity and expression of anti-cancer proteins. International Journal of Immunopathology and Pharmacology, 21, 117-127.

Li, Q., Morimoto, K., Kobayashi, M., Inagaki, H., Katsumata, M., Hirata, Y., ... & Miyazaki, Y. (2008b). A forest bathing trip increases human natural killer activity and expression of anti-cancer proteins in female subjects. Journal of Biological Regulators and Homeostatic Agents, 22, 45-55.

Li, Q., Morimoto, K., Nakadai, A., Inagaki, H., Katsumata, M., Shimizu, T., ... & Kawada, T. (2007). Forest bathing enhances human natural killer activity and expression of anti-cancer proteins. International Journal of Immunopathology and Pharmacology, 20(2 Suppl 2), 3-8.

Liu GC, Wilson JS., Qi R., Ying, J. (2007). Green Neighborhoods, Food Retail and Childhood Overweight: Differences by Population Density. American Journal of Health Promotion; 21(4 Suppl): 317-325.

Loughner CP, Allen DJ, Zhang DL, Pickering KE, Dickerson RR, & Landry L. (2012). Roles of urban tree canopy and buildings in Urban Heat Island effects: Parameterization and Preliminary Results. J Applied Meteorology Climatology, 51:1775-1793. doi: 10.1175/JAMC-D-11-0228.1

Lovasi GS, Jacobson JS, Quinn JW, Neckerman KM, Ashby-Thompson MN, Rundle A. (2011). Is the environment near home and school associated with physical activity and adiposity of urban preschool children? Journal of Urban Health, 88, 1143-1157. doi: 10.1007/s11524-011-9604-3.

Lovasi GS, O’Neil-Dunne JP, Lu JW, Sheehan D, Perzanowski MS, et al. 2013. Urban tree canopy and asthma, wheeze, rhinitis, and allergic sensitization to tree pollen in a New York City birth cohort. Environ. Health Perspect. 121:494–500, 00e1–6

Lovasi, G. S., Bader, M. D., Quinn, J., Neckerman, K., Weiss, C., & Rundle, A. (2012). Body mass index, safety hazards, and neighborhood attractiveness. American Journal of Preventive Medicine, 43, 378-384.

Lovasi, G. S., Quinn, J. W., Neckerman, K. M., Perzanowski, M. S., & Rundle, A. (2008). Children living in areas with more street trees have lower prevalence of asthma. Journal of Epidemiology and Community Health, 62, 647-649.

Maas, J., Verheij, R. A., Spreeuwenberg, P., & Groenewegen, P. P. (2008). Physical activity as a possible mechanism behind the relationship between green space and health: a multilevel analysis. BMC Public Health, 8, 206.

Markevych, I., Thiering, E., Fuertes, E., Sugiri, D., Berdel, D., Koletzko, S., ... & Heinrich, J. (2014a). A cross-sectional analysis of the effects of residential greenness on blood pressure in 10-year old children: results from the GINIplus and LISAplus studies. *BMC Public Health*, *14*, 477.

Markevych, I., Tiesler, C.M.T., Fuertes, E., Romanos, M., Dadvand, P., Nieuwenhuijsen, M.J., Berdel, D., Koletzko, S., Heinrich, J. Access to urban green spaces and behavioural problems in children: Results from the GINIplus and LISAplus studies. (2014b). Environment International, 71, pp. 29-35.

Michimi A & Wimberly MC. (2012). Natural Environments, Obesity, and Physical Activity in Nonmetropolitan Areas of the United States. The Journal of Rural Health 28, 398–407. doi: 10.1111/j.1748-0361.2012.00413.x

Miles R, Coutts C, Mohamadi A. (2012). Neighborhood urban form, social environment, and depression. J Urban Health 89(1):1-18. doi: 10.1007/s11524-011-9621-2.

Mitchell, R., & Popham, F. (2008). Effect of exposure to natural environment on health inequalities: an observational population study. The Lancet, 372, 1655-1660.

Miyazaki Y, Ikei H, Song C. (2014). Forest medicine research in Japan. Nihon eiseigaku zasshi. Japanese journal of hygiene 69:2, 122-35.

Nakamura, R. & Fujii, E. (1990). Studies of the characteristics of the electroencephalogram when observing potted plants: Pelargonium hortorum “Sprinter red” and begonia evansiana. *Tech. Bull. Fac. Horticult. Chiba Univ.* **1990**, *43*, 177–183.

Nakamura, R. & Fujii, E. (1992). A comparative study of the characteristics of the electroencephalogram when observing a hedge and a concrete block fence. *J. Jpn. Inst. Landsc. Archit.*, *55*, 139–144.

Nutsford D, Pearson AL, Kingham S. (2013). An ecological study investigating the association between access to urban green space and mental health. Public Health 127, 1005-1011. doi: 10.1016/j.puhe.2013.08.016

Paoletti E, Bardelli T, Giovannini G, & Pecchioli L. (2011). Air quality impact of an urban park over time. Procedia Environ. Sci. 4:10–16

Park, B.J., Tsunetsugu, Y., Kasetani, T., Hirano, H., Kagawa, T., Sato, M., & Miyazaki, Y. (2007). Physiological effects of Shinrin-yoku (taking in the atmosphere of the forest)-using salivary cortisol and cerebral activity as indicators. Journal of Physiological Anthropology, 26, 123-128.

Park, B.J., Tsunetsugu, Y., Kasetani, T., Kagawa, T., & Miyazaki, Y. (2010). The physiological effects of Shinrin-yoku (taking in the forest atmosphere or forest bathing): evidence from field experiments in 24 forests across Japan. Environmental Health and Preventive Medicine, 15, 18-26.

Paternostro-Bayles M, Wing RR, Robertson RJ (1989) Effect of life-style activity of varying duration on glycemic control in type II diabetic women. Diabetes Care 12:34–37.

Pereira G, Christian H, Foster S, Boruff BJ, Bull F, Knuiman M, Giles-Corti B. (2013). The association between neighborhood greenness and weight status: an observational study in Perth Western Australia. Environ Health 12:49.

Pereira, G., Foster, S., Martin, K., Christian, H., Boruff, B. J., Knuiman, M., & Giles-Corti, B. (2012). The association between neighborhood greenness and cardiovascular disease: an observational study. BMC public health, 12, 466. doi: 10.1186/1471-2458-12-466.

Perry H, Davies HW, Frank L, Van Loon J, Gehring U, Tamburic L, & Brauer M. (2014). Residential Greenness and Birth Outcomes: Evaluating the Influence of Spatially Correlated Built-Environment Factors. Environmental Health Perspectives, 122(10):1095-1102. doi: 10.1289/ehp.1308049

Pilat MA, McFarland A, Snelgrove A, Collins K, Waliczek TM, Zajicek J. 2012. The effect of tree cover and vegetation on incidence of childhood asthma in metropolitan statistical areas of Texas. Horttechnology 22(5):631–637.

Potwarka LR1, Kaczynski AT, & Flack AL. (2008). Places to play: association of park space and facilities with healthy weight status among families. J Community Health 33, 344-50. doi: 10.1007/s10900-008-9104-x.

Poudyal, N. C., Hodges, D. G., Bowker, J. M., & Cordell, H. K. (2009). Evaluating natural resource amenities in a human life expectancy production function. Forest Policy and Economics, 11, 253-259.

Richardson, E., Pearce, J., Mitchell, R., Day, P., & Kingham, S. (2010). The association between green space and cause-specific mortality in urban New Zealand: an ecological analysis of green space utility. BMC Public Health,10, 240.

Richardson, EA & Mitchell, R. 2010. Gender differences in relationships between urban green space and health in the United Kingdom. Social Science & Medicine, 71(3), 568-575. doi: 10.1016/j.socscimed.2010.04.015

Richardson, EA, Mitchell, R., Hartig, T., de Vries, S., Astell-Burt, T., & Frumkin, H. (2011). Green cities and health: a question of scale? Journal of Epidemiology and Community Health, 66, 160-165.

Rook, G.A. (2013). Regulation of the immune system by biodiversity from the natural environment: An ecosystem service essential to health. Proceedings of the National Academy of Sciences, 110, 18360-18367.

Ryushi T, Kita I, Sakurai T, Yasumatsu M, Isokawa M, Aihara Y, Hama K. The effect of exposure to negative air ions on the recovery of physiological responses after moderate endurance exercise. Int J Biometeorol 1998;41: 132–6.

Schneider SH, Khachadurian AK, Amorosa LF, Gravras H, Fineberg SE, Ruderman NB (1987) Abnormal glucoregulation during exercise in type II (non-insulin-dependent) diabetes. Metabolism 36:1161–1166.

Segerstrom SC, & Miller, GE. (2004). Psychological stress and the human immune system: A Meta-Analytic Study of 30 Years of Inquiry. Psych Bull, 130(4), 601-630. doi: 10.1037/0033-2909.130.4.601

Seo, D., Patrick, C.J., & Kennealy, P.J. (2008). Role of Serotonin and Dopamine System Interactions in the Neurobiology of Impulsive Aggression and its Comorbidity with other Clinical Disorders. *Aggression and Violent Behavior*, *13*(5), 383–395. doi:10.1016/j.avb.2008.06.003

Simpson KA, Singh MA (2008) Effects of exercise on adiponectin: a systematic review. Obesity 16:241–256

Song, C., Ikei, H., Igarashi, M., Miwa, M., Takagaki, M., & Miyazaki, Y. (2014). Physiological and psychological responses of young males during spring-time walks in urban parks. Journal of Physiological Anthropology, 33, 8.

Sullivan WC, Kuo FE, & DePooter SF. (2004). The fruit of urban nature: Vital neighborhood spaces. Environment & Behavior, 36(5), 678-700.

Suzuki S, Yanagita S, Amemiya S, Kato Y, Kubota N, Ryushi T, Kita I. (2008). Effects of negative air ions on activity of neural substrates involved in autonomic regulation in rats. Int J Biometeorol 52(6):481-9. Epub 2008 Jan 11.

Takano, T., Fu, J., Nakamura, K., Uji, K., Fukuda, Y., Watanabe, M., & Nakajima, H. (2002). Age-adjusted mortality and its association to variations in urban conditions in Shanghai. *Health Policy*, *61*, 239-253.

Takano, T., Nakamura, K., & Watanabe, M. (2002). Urban residential environments and senior citizens’ longevity in megacity areas: the importance of walkable green spaces. Journal of Epidemiology and Community Health, 56, 913-918.

Tamosiunas, A., Grazuleviciene, R., Luksiene, D., Dedele, A., Reklaitiene, R., Baceviciene, M., ... & Nieuwenhuijsen, M. J. (2014). Accessibility and use of urban green spaces, and cardiovascular health: findings from a Kaunas cohort study. Environmental Health, 13(1), 20.

Tilt JH, Unfried TM, Roca B. Using objective and subjective measures of neighborhood greenness and accessible destinations for understanding walking trips and BMI in Seattle, Washington. Am J Health Promot. 2007;21(4 Suppl):371–9. [PubMed]

Troy, A. R., Morgan Grove, J., & O’Neil-Dunne, J. (2012). The relationship between tree canopy and crime rates across an urban–rural gradient in the greater Baltimore region. Landscape and Urban Planning, 106(3), 262–270. doi:10.1016/j.landurbplan.2012.03.010

Ulrich RS, Simons RF, Losito BD, Fiorito E, Miles MA, Zelson M. (1991). Stress recovery during exposure to natural and urban environments. Journal of Environmental Psychology 11, 201-230.

Van den Berg, A. E., M. Van Winsum-Westra, S. De Vries, and S. Van Dillen. 2010. Allotment gardening and health: A comparative survey among allotment gardeners and their neighbors without an allotment. Environmental Health 9:74.

Van den Berg, A.E., van den Berg, C.G. (2011). A comparison of children with ADHD in a natural and built setting. Child: Care, Health and Development, 37(3), 430-439.

Villeneuve PJ, Jerrett M, Su JG, Burnett, RT, Chen H, Wheeler AJ, & Goldberg MS. (2012). A cohort study relating urban green space with mortality in Ontario, Canada. Environmental Research 115:51-58.

von Hertzen L, Hanski I, Haahtela T (2011) Natural immunity: Biodiversity loss and inflammatory diseases are two global megatrends that might be related. EMBO Rep 12:1089–1093.

Ward Thompson, C., Roe, J., Aspinall, P., Mitchell, R., Clow, A., & Miller, D. (2012). More green space is linked to less stress in deprived communities: Evidence from salivary cortisol patterns. Landscape and Urban Planning,105, 221-229.

Weinstein N, Przybylski AK, & Ryan RM. (2009). Can Nature Make Us More Caring? Effects of Immersion in Nature on Intrinsic Aspirations and Generosity. Personality and Social Psychology Bulletin, 35(10): 1315-1329.

Wells NM, & Evans GW. (2003). Nearby nature: A buffer of life stress among rural children. Environment and Behavior, 35(3), 311-330. doi:  10.1177/0013916503251445

Wilker, EH, Wu, CD, McNeely, E, Mostofsky, E, Spengler, J, Wellenius, GA, & Mittleman, MA (2014). Green space and mortality following ischemic stroke. Environmental Research, 133, 42-48.

Wolch, J., Jerrett, M., Rynolds, K., McConnell, R., Chang, R., Dahmann, N., Brady, K., Gilliland, F., Su, JG., Berhane, K. (2011). Childhood obesity and proximity to urban parks and recreational resources: a longitudinal cohort study. Health & Place; 17: 207-214. doi:10.1016/j.healthplace.2010.10.001

Wolfe, MK & Mennis, J. (2012). Does vegetation encourage or suppress urban crime? Evidence from Philadelphia, PA. Landscape and Urban Planning. doi:10.1016/j.landurbplan.2012.08.006

Yoo, MH; Kwon, YJ; Son, KC & Kays,SJ. (2006). Efficacy of indoor plants for the removal of single and mixed volatile organic pollutants and physiological effects of the volatiles on the plants. J. Am. Soc. Hort. Sci. 131:452–58.
